# Morphological and Molecular Characterization of Three Myxosporean Species of the Genera *Myxobolus*, *Henneguya*, and *Myxidium* (Cnidaria: Myxozoa) Infecting Freshwater Fish, Isolated for the First Time in Japan

**DOI:** 10.3390/life14080974

**Published:** 2024-08-02

**Authors:** Mariko Sekiya, Haruya Sakai, Ying-Chun Li, Imron Rosyadi, Muchammad Yunus, Hiroshi Sato

**Affiliations:** 1 Joint Faculty of Veterinary Medicine, Yamaguchi University, Yamaguchi 753-8515, Japan; 2 Joint Graduate School of Veterinary Medicine, Yamaguchi University, Yamaguchi 753-8515, Japan; lyliyingchun2010@yahoo.co.jp (Y.-C.L.); imron.rosyadi@ugm.ac.id (I.R.); 3 Faculty of Veterinary Medicine, Airlangga University, Mulyorejo, Surabaya 60115, Indonesia; muhyunus_99@yahoo.com

**Keywords:** *Myxobolus tribolodonus* sp. n., *Henneguya pungitii*, *Myxidium salvelini*, freshwater fish, morphology, small subunit ribosomal RNA gene (SSU rDNA), Japan

## Abstract

The majority of myxosporean species (Cnidaria: Myxozoa) of the genera *Myxobolus* (35 species), *Henneguya* (8 species), and *Myxidium* (9 species) from freshwater or brackish fish in Japan were recorded more than 30 years ago (accumulatively 81.1% [43/53]). The re-discovery and molecular–genetic characterization of these species is a current research priority. During our myxosporean survey in Japanese freshwater fish, we detected three species that had never been recorded in Japan, but in the Russian Far East (Sakhalin Island, and Maritime Province): *Myxobolus tribolodonus* sp. n., forming cysts in the gills of *Tribolodon sachalinensis* (syn. *M. marinus* sensu Aseeva, 2000; *M. marinus* sensu Sokolov et Frolova, 2015, recorded from the gills of *Pseudaspius* (syn. *Tribolodon*) spp.); *Henneguya pungitii* Achmerov, 1953, forming cysts in the subcutis of external skin and buccal submucosa of *Pungitius sinensis*; and *Myxidium salvelini* Konovalov et Shulman, 1966, in the urinary bladder of *Oncorhynchus masou ishikawae*. These new isolates were characterized by integrated taxonomic approaches, i.e., myxospore morphology and molecular–genetic characterization of the small subunit ribosomal RNA gene (SSU rDNA). These new isolates were phylogenetically differentiated from any species whose SSU rDNA sequences were deposited in the DNA databases, and concurrently compared with recorded species based on classical morphological criteria. All three species were differentiated from myxosporeans previously recorded in Japan, indicating new distribution records out of the Russian Far East. For reliable species identification, accumulation of at least SSU rDNA sequences of known species worldwide is critically important.

## 1. Introduction

Endoparasitic cnidarians of the subclass Myxosporea Bütschli, 1881 (phylum Cnidaria Hatschek, 1888: class Myxozoa Grassé, 1970), take invertebrate (annelids) and vertebrate hosts (mainly fish) alternatively in their life cycles in aquatic environment [1]. They are often incriminated as causatives of a variety of symptomatic or moribund diseases of farmed fish, causing significant economic damage to the aquaculture and fishery industries [2,3,4]. Except for such symptomatic cases, myxosporean infection is generally latent, with patchy spatial distribution, and geographical knowledge of myxozoans can be biased, reflecting predilections of investigators. There are few incentives to investigations on parasites of fishes, as they have little commercial value [5]. Nevertheless, more than 2600 myxozoan species have been described, and extensive uncharted biodiversity of myxozoans remains to be investigated [5,6,7].

Two orders—Bivalvulida Shulman, 1959, and Multivalvulida Shulman, 1959—are divided in the subclass Myxosporea based on the numbers of shell valves (SVs) and polar capsules (PCs) in a myxospore [6,8]. Members of Bivalvulida are characterized by two SVs and two PCs in a myxospore (exceptionally, four PCs in a myxospores with two SVs in the genus *Chloromyxum* Mingazzini, 1890), and currently, 57 genera are classified in Bivalvulida [6,8]. The most speciose genus, *Myxobolus* Bütschli, 1882, contains more than 970 nominal species [8,9,10,11]. The genera *Ceratomyxa* Thélohan, 1892; *Myxidium* Bütschli, 1882; *Henneguya* Thélohan, 1892; *Chloromyxym*; and *Thelohanellus* Kudo, 1933 are also speciose, counting more than 270, 230, 210, 140, and 100 species, respectively [6,12,13,14,15,16,17,18,19,20].

Approximately sixty myxosporean species of the Bivalvulida have been recorded from freshwater and brackish fish in Japan, but most of them were recorded more than 30 years ago when specific descriptions were made solely based on myxospore morphology. For example, among nominal species of the genera *Myxobolus* (35 species), *Henneguya* (9 species), and *Myxidium* (9 species), 49.1% (26/53) were recorded more than 90 years ago and 81.1% (43/53) more than 30 years ago, as shown in Supplementary Table S1. Furthermore, the majority of these species have never been re-isolated or re-characterized with modern taxonomic viewpoints, i.e., specific characterization based on myxospore morphology and nucleotide sequence of the ribosomal RNA gene (rDNA), as has been recommended recently [21]. To date, only 37.7% (20/53) of species of the aforementioned three genera in Japan have been characterized using molecular–genetic techniques (Supplementary Table S1).

To address the taxonomic issues mentioned above, we are conducting a survey of myxosporean infection in freshwater fish in Japan. In the present study, we characterize three myxosporean species of the genera *Myxobolus*, *Henneguya,* and *Myxidium* and compare them with previously recorded species from Japan and other regions, particularly in the Russian Far East and China, where multiple myxosporean species recorded in freshwater and brackish fishes are shared with Japan [22,23,24].

## 2. Materials and Methods

### 2.1. Fish Samples

The World Fresh Water Aquarium Gifu (Aquatotto Gifu) in Kakamigahara City, Gifu Prefecture, Japan, which exhibits approximately 28,500 freshwater fish of 260 species worldwide, regularly collects native Japanese fish species for exhibition. After careful acclimatization in aquarium water, these introduced fish are transferred to exhibition tanks. Surveillance checks for dead fish are carried out daily and the dead specimens are subsequently frozen and provided to us, as described previously [25]. For this study, the bodies of 92 dead fish of 34 species (13 families) were examined between 10 October 2017 and 22 September 2018 (Supplementary Table S2).

### 2.2. Parasitological Examination

The frozen fish bodies were thawed, and the body weights and total and standard body lengths were recorded. The external and internal organs were then individually examined with the naked eye and under a dissection microscope. To detect myxosporeans microscopically, the contents of the luminal organs, such as the gallbladder, urinary bladder, and gastrointestinal tract, were smeared on glass slides and treated with Diff-Quik™ stain (Sysmex Co., Kobe, Japan).

Myxosporean cysts found in the gills and subcutis/submucosa were opened using fine forceps in physiological saline. Similarly, the contents of the urinary bladder containing myxospores were diluted with physiological saline. These were then observed using a light microscope (OLYMPUS BX60; OLYMPUS Co., Hachioji, Tokyo, Japan) equipped with differential interference contrast optics, photographed at a magnification of ×800, then transformed into digital images using Adobe^®^ Photoshop^®^ ver. 11.0 (Adobe Systems, San Jose, CA, USA). The photographs were then printed at a high magnification. Measurements were conducted on multiple printed photographs following the guidelines of Lom and Arthur [26]. All measurements are expressed in micrometers (µm) unless otherwise stated. Ranges with the means in parentheses are presented. Following the removal of a portion of the myxospores for DNA extraction, the parasite was fixed in either 10% neutral-buffered formalin solution or 70% ethanol solution. The specimens collected in the present work were deposited in the Meguro Parasitological Museum, Tokyo, Japan, under collection nos. 21656–21658.

### 2.3. DNA Extraction, Polymerase Chain Reaction (PCR), and Sequencing

Parasite DNA was extracted from collected myxospores using an Illustra™ tissue and cells genomicPrep Mini Spin Kit (GE Healthcare UK, Buckinghamshire, UK) according to the manufacturer’s instructions. PCR amplification of the small subunit (SSU) rDNA was performed in a 20 µL volume containing a DNA polymerase, Blend Taq-Plus- (TOYOBO, Dojima Hama, Osaka, Japan), and a combination of universal eukaryotic primers, Eurib1 (5′-ACCTGGTTGATCCTGCCAG-3′) and reverse Eurib2 (5′-CTTCCGCTGGTTCACCTACGG-3′), was used to amplify almost the complete length of the SSU rDNA at once [27,28]. The PCR products were purified using a FastGene Gel/PCR Extraction Kit (NIPPON Genetics Co., Tokyo, Japan), and the purified PCR products were cloned into the plasmid vector pTA2 (TArget Clone™; TOYOBO) and transformed into *Escherichia coli* JM109 cells (TOYOBO) according to the manufacturer’s instructions. Following propagation, the plasmid DNA was extracted using a FastGene Plasmid Mini Kit (NIPPON Genetics Co., Tokyo, Japan), and inserts from multiple independent clones, at least three, were sequenced using universal M13 forward and reverse primers (5′-GTAAAACGACGGCCAGT-3′; and 5′-GGAAACAGCTATGACCATG-3′, respectively). For sequencing purposes, some additional primers were chosen from our stocks used in the previous works [25,28,29]; NSR581/18 (5′-TCTCAGGCTCCCTCTCCGG-3′), Myxo18S_794R (5′-CGCCTGCTTTGAGCACTCTGT-3′), Myxo18S_1009R (5′-CGCATCTGTTAGTCCTTGG C-3′), Myxo 18S_575F (5′-CGCGGTAATTCCAGCTCCAG-3′), Myxo18S_887F (5′-AATGG TCGAGGGCAACTTTG-3′), Myxo18S_1028F (5′-GCCAAGGACTAACAGATGCG-3′), or Myxo18S_1217F (5′-GGGAGAGTATGGTCGCAAGT-3′).

The nucleotide sequence obtained in the present study is available from the DDBJ/EMBL/GenBank databases under accession nos. LC544125–LC544127.

### 2.4. Phylogenetic Analysis

Fragments of the newly obtained rDNA sequences were analyzed to identify highly similar nucleotide sequences using the Basic Local Alignment Search Tool (BLAST) of the National Center for Biotechnology Information website (NCBI; https://www.ncbi.nlm.nih.gov/ (accessed on 7 July 2020). For phylogenetic analysis, the newly obtained SSU rDNA nucleotide sequences in the present study and related myxosporean sequences retrieved from the DDBJ/EMBL/GenBank databases were aligned using the MEGA7 software [30], with subsequent manual adjustments. The accession numbers of the sequences analyzed in the present study are provided in the figure showing a phylogenetic tree. Regions judged to be poorly aligned and characters with a gap in any sequence were excluded; 920 characters, of which 416 were variable, were retained for subsequent analysis. Maximum likelihood (ML) analysis was performed using the PhyML program ver. 3.0 [31,32] provided on the ‘phylogeny.fr’ website (http://www.phylogeny.fr/). The probability of inferred branch was assessed by the approximate likelihood-ratio test (aLRT), an alternative to the non-parametric bootstrap estimation of branch support [33].

## 3. Results

**Figure 1:**
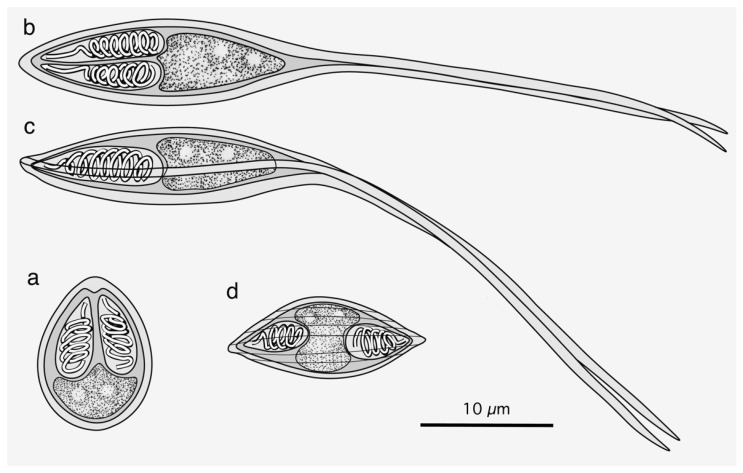
Stylized illustrations of *Myxobolus tribolodonus* n. sp. from *Pseudaspius sachalinensis* (**a**), *Henneguya pungitii* from *Pungitius sinensis* (**b**,**c**), and *Myxidium salvelini* from *Oncorhynchus masou* (**d**). Frontal view (**a**,**b**,**d**) and sutural view (**c**) at the same magnification.

Three myxosporean species of the genera *Myxobolus*, *Henneguya*, and *Myxidium* were found separately in three fish species (Figure 1). Four *Myxobolus* plasmodia were found in the gills of a rosyface dace, *Pseudaspius sachalinensis* (Nikolskii, 1889) (syn. *Tribolodon sachalinensis* (Nikolskii, 1889)), measuring 11.0 cm in standard body length (BL) and 20.6 g body weight (BW), collected originally in May 2018 from a river running through Akkeshi-cho Town, Hokkaido Prefecture, Japan. Four cysts at the subcutis of the external surface in the anterior body and more than 20 cysts in the oral submucosa were found in an Amur stickleback, *Pungitius sinensis* (Guichenot, 1869), measuring 4.9 cm BL and 1.2 g BW, collected originally in May 2018 from the same river running through Akkeshi-cho Town. These cysts contained *Henneguya* plasmodia. Numerous *Myxidium* myxospores were found in the urinary bladder of a masu salmon, *Oncorhynchus masou* (Brevoort, 1856) (syn. *Oncorhynchus masou ishikawae* (Jordan et McGregor, 1925)), measuring 30.5 cm BL and 599 g BW, collected originally in May 2018 from the Nagaragawa River running through Gifu Prefecture, Japan. Since the myxospore morphology and SSU rDNA nucleotide sequences of the aforementioned species were differentiated from previously recorded species in Japan, we carefully conducted their specific identification with species recorded from other locations.

### 3.1. Myxobolus tribolodonus sp. n. (Myxosporea: Bivalvulida: Myxobolidae)

**Figure 2:**
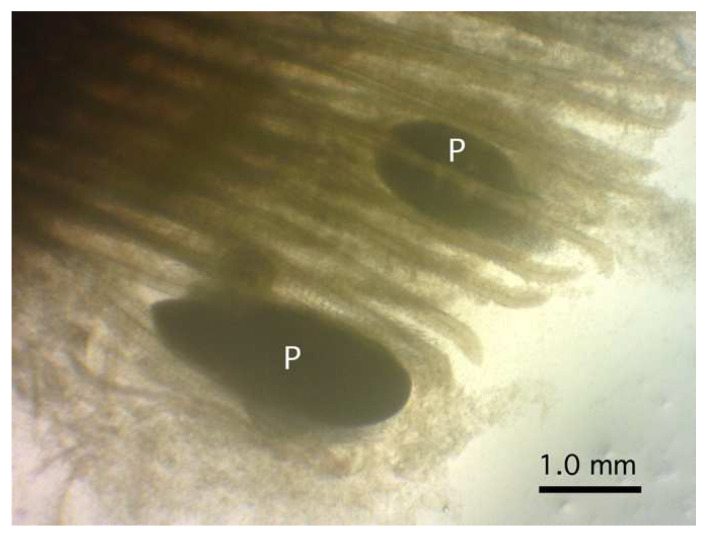
Plasmodia (P) of *Myxobolus tribolodonus* n. sp. attached to the gill filaments of *Pseudaspius sachalinensis* under a dissection microscope.

**Figure 3:**
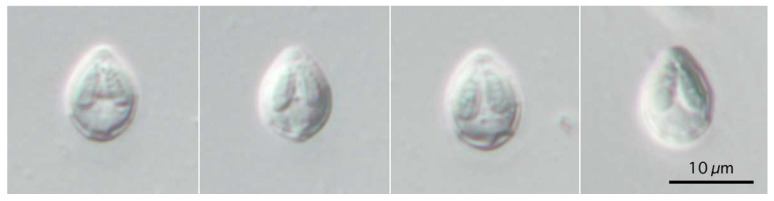
Photomicrographs of myxospores of *Myxobolus tribolodonus* n. sp. from *Pseudaspius sachalinensis.* Frontal view at the same magnification.

(syn. *Myxobolus marinus* sensu Aseeva, 2000; *M. marinus* sensu Sokolov et Frolova, 2015)

(Figure 1a, Figure 2 and Figure 3)

Four large-sized oval plasmodia were detected between the gill filaments at their upper half portion of a rosyface dace (Figure 2). The largest plasmodium measured 2.35 mm by 0.87 mm, and the other three were smaller, measuring 1.13–1.29 (1.24) mm by 0.44–0.77 (0.64) mm.

#### 3.1.1. Description

Bivalvular myxospores pyriform from the frontal view, measuring 8.7–9.6 (9.2) in length, 6.5–7.5 (7.0) in width (n = 11). Valvular surface smooth. Two almost equal PCs, bullet-shaped, 4.6–6.0 (5.1) in length and 1.7–2.1 (1.9) in width, pointing towards the apical end, and the posterior end of PCs beyond the mid-line of myxospore length. Binucleated sporoplasm in the remaining space. A small-sized intercapsular appendix, and 4–5 turns of polar tubules.

#### 3.1.2. Molecular–Genetic Characterization

A newly obtained, almost complete SSU rDNA nucleotide sequence was 2007 bp in length (DDBJ/EMBL/GenBank accession no. LC544125), and showed the highest identities (up to 95.11% [1538/1617]) with sequences from certain *Myxobolus* spp. such as *M. alvarezae* (accession no. FJ716097), *M. intimus* (accession no. FJ716098), *M. sitjae* (accession no. JF311898), *M. obesus* (accession no. AY325286), and *M. szentendrensis* (accession no. KP025684), which had 5–11 nucleotide insertion/deletion sites (indels) over an rDNA sequence comparable with that of the new species. The nucleotide identity of the SSU rDNA sequence of *Myxobolus macrocapsularis* Reuss, 1906, from *Abramis brama* (L., 1758) or *Blicca bjoerkna* (L., 1758) in Hungary (accession nos. AF507969 and FJ716095, respectively) with that of the present new species was 83.58% (1308/1565) with 33 indels, or 81.01% (1058/1306) with 27 indels. Shulman [22] considered *M. macrocapsularis* as a senior synonym of *M. marinus* Dogiel, 1948, from the gills of *Alburnus alburnus* (L., 1758) in the Russian Far East (Amur River basin and other rivers flowing to the Sea of Japan).

#### 3.1.3. Remarks

Myxospores of this myxobolid species were pyriform, with a length of 8–10 µm, apparently classified in the genus *Myxobolus* according to Lom and Dyková [6]. More than 40% of ca. 970 nominal *Myxobolus* spp. showed an organ/tissue preference for the gills [9,10,11]. The morphologically identical myxospores were recorded from *P*. *sachalinensis* (type host of the present new species), *Pseudaspius hakonensis* (Günther, 1877) (syn. *Tribolodon hakonensis* (Günther, 1877)), and *Pseudaspius brandtii* (Dybowski, 1872) (syn. *Tribolodon brandtii* (Dybowski, 1872)) from the rivers on the Sakhalin Island or in the Maritime Province (Primorsky Kray) of the Russian Far East [34,35], as shown in Table 1. These isolates (*M. tribolodonus* sp. n., *M. marinus* sensu Aseeva, 2000, and *M. marinus* sensu Sokolov et Frolova, 2015) had identical myxospore dimensions and proportions (myxospore length/width), with an index of approximately 1.2 (1.1–1.3). *Myxobolus marinus* Dogiel, 1948, was originally described from the gill lamellae of *Alburnus alburnus* (Cypriniformes: Leuciscidae: Leuciscinae) in the basin of the Amur River and the Sea of Japan [22,36,37], and is currently considered to be a junior synonym of *Myxobolus macrocapsularis* [22,37]. The dimensions of *M. macrocapsularis* myxospores, including the original description of *M. marinus,* are apparently larger than the myxospores found in *Pseudaspius* spp. in the Russian Far East region (Maritime Province (Primorsky Kray) and Sakhalin Island) and Hokkaido Island of Japan (Table 1). Although Shulman [22] characterized myxospores of the former species as pyriform with narrow and pointed anterior ends, none of the isolates from the gills of *Pseudaspius* spp. (*M. tribolodonus* sp. n., *M. marinus* sensu Aseeva, 2000, and *M. marinus* sensu Sokolov et Frolova, 2015) showed such traits, but they showed comparatively bluntly pointed anterior ends of pyriform myxospores (Figure 1a and Figure 3 in the present study; also see [34,35]). Ellipsoidal plasmodia of *M. macrocapsularis* and *M. tribolodonus* sp. n. exhibited almost similar dimensions and localization near the tips of the gill filaments (Figures 3–5 in [37]; Figure 2 of the present study).

Further morphological comparisons with nominal *Myxobolus* spp. were conducted with 17 *Myxobolus* spp. (Supplementary Table S3), selected based on the myxospore shape (non-circular in the frontal view) and the myxospore length of between 8–10 µm using synoptic references [9,10,11]. Only four species (*M. bengalensis*, *M. branchialis*, *M. flavus*, and *M. tambroides*) have deposited SSU rDNA nucleotide sequences (DDBJ/EMBL/GenBank accession nos. KJ476883, MK412934, JQ388887, KF296346, KF296347, and JX028236), and these sequences show less than 90% identity with that of *M. tribolodonus* sp. n. (accesssion no. LC544125), in addition to a few to several indels within the 1520 bp to 1935 bp long sequences. Morphologically, the PCs of *M. bengalensis*, *M. branchialis*, *M. flavus*, and *M. tambroides* are wider than those of the present species (Supplementary Table S3) and situated closer to each other within a myxospore, in contrast to the distantly placed PCs in *M. tribolodonus* n. sp. For the remaining 13 species, no nucleotide sequences are available in the DNA databases. All of these species, except for *M. funsienensis* (Ma, 1998) Erias et al., 2005 (syn. *Myxosoma fusienenesis* Ma in Chen et Ma, 1998), differed apparently from *M. tribolodonus* sp. n. in their elliptical myxospore shape, wider PCs, smaller angle of two PCs, number of turns of polar tubules, and/or small dimensions of cysts (Supplementary Table S3). Although histological localization of cysts in the gills is known to have high importance in species identification [38], such data are not always available. The present new species shows similar myxospore morphology to that of *M. fusienensis* recorded in the gills of *Spinibarbichthys yunnanensis* (Tsü, 1977) (syn. *Spinbarbus denticulatus yunnanensis* (Tsü, 1977)) from the southernmost province in China (Yunnan), but these two species differ from each other in the shape of PCs (pyriform vs. bullet-shaped), the angular arrangement of PCs (wide vs. narrow), and the absence and presence of small intercapsular processes [9,23].

**Table 1:** Comparison of *Myxobolus tribolodonus* sp. n. with its related species ^a^.

Species	Host Fish	Location in Host	Locality	SL	SW	ST	PCL	PCW	SF	IP	NT	Cyst Size	Reference
*M. tribolodonus* sp. n.	*Pseudaspius sachalinensis* (syn. *Tribolo-don sachalinensis*)	Gills	Japan (Hokkaido)	8.7–9.6 (9.2)	6.5–7.5 (7.0)	—	4.6–6.0 (5.1)	1.7–2.1 (1.9)	Pyriform	Small	4–5	max. 2.35 by 0.87 mm	Present Study
*M. marinus* sensu Aseeva, 2000	*Pseudaspius brandtii* (syn. *Tribolodon brandtii*); *Pseudaspius hakonensis* (syn. *Tribolodon hakonensis*)	Gills	Maritime Province (Primorsky Kray) of Russian Far East	9.7–12.0	8.3–10.3	—	5.5–6.3	—	pyriform	—	—	—	[34]
*M. marinus* sensu Sokolov et Frolova, 2015	*Pseudaspius sachalinensis* (syn. *Tribolodon sachalinensis*); *Pseudaspius hakonensis* (syn. *Tribolodon hakonensis*)	Gills	Sakhalin Island (Russian Far East)	9.9–11.3 (10.7)	8.1–9.2 (8.6)	—	5.0–6.1 (5.7)	2.5–3.4 (3.0)	pyriform	small	—	—	[35]
*M. marinus* Dogiel, 1948	*Alburnus alburnus*	Gills	The basin of the Amur River and the Sea of Japan	12.5–13.0	7.0–8.0	—	6.0–7.0	2.5–3.0	pyriform	small	—	—	[22,36]
*M. macrocapsularis* (syn. *Myxobolus physophilus* Reuss, 1906; *M. multiplex* Achmerov, 1960; *M. vescus* Achmerov, 1960; *M. oviformis* Thélohan in Rostovschikov, 1952; *M. marinus* Dogiel, 1948) ^b^	*Rutilus rutilus* (L., 1758) ; *Leu-ciscus leuciscus* (L., 1758); *Leu-ciscus idus* (L., 1758); *Squalius cephalus* (L., 1758) (syn. *Leuci-scus cephalus* (L., 1758); *Scardi-nius erythrophthalmus* (L., 1758); *Leuciscus aspius* (L., 1758) (syn. *Aspius aspius* (L., 1758); *Barbus barbus* (L., 1758); *Gobio gobio* (L., 1758) ; *Abbottina rivularis* (Basilewsky, 1855) (syn. *Pseudo-gobio rivularis* (Basilewsky, 1855)); *Alburnus alburnus*; *Alburnoides bipunctatus* (Bloch, 1782) (syn. *Alburnus bipunctatus* (Bloch, 1782)); *Chondrostoma nasus* (L., 1758); *Pelecus cultratus* (L., 1758): *Opsariichthys uncirostris* (Temminck et Schlegel, 1846); *Carassius carassius* (L., 1758); *Cyprinus carpio* L., 1758; *Hypophthalmichthys molitrix* (Valenciennes, 1844) ; *Gasterosteus aculeatus* L., 1758	Gills, mesentery, intestinal wall, swimbladder	Basins of rivers in southern Karelia, Rivers flowing to the Baltic Sea, Rivers flowing to the Azov Sea, or Caspian Sea; and Amur River,	9.0–14.5	6.0–9.5	4.5–6.0	5.0–8.6	2.4–3.6	pyriform	small	—	Reaching 1.5 mm in diameter	[22]

^a^ Abbreviation: SL, myxospore length; SW, myxospore width; ST, myxospore thickness; PCL, polar capsule length; PCW, polar capsule width; SF, myxospore form; IP, intercapsular projection; NT, number of coil turns (polar tubles). All measurements are expressed in micrometers (µm) unless otherwise stated. Ranges are presented, with the means in parentheses. For *M. flavus*, means and standard variations are shown in parentheses. ^b^ All synonyms follow Shulman (1966).

#### 3.1.4. Taxonomic Summary

- Host: *Pseudaspius sachalinensis* (syn. *Tribolodon sachalinensis*), rosyface dace (Actinopterygii: Cypriniformes: Leuciscidae: Pseudaspininae).
- Additional hosts: *Pseudaspius hakonensis* (syn. *Tribolodon hakonensis*) and *Pseudaspius brandtii* (syn. *Tribolodon brandtii*) [34,35].
- Type locality: Collected in Aquatotto Gifu, Gifu Prefecture, Japan, following its relocation from a river running through Akkeshi-cho Town, Hokkaido Prefecture, Japan. Circumstantially, the infection might originate from the water in the natural habitat and not in the aquarium.
- Additional locality: Rivers on the Sakhalin Island or in the Maritime Province (Primorsky Kray) of Siberia (Russia) [34,35].
- Site of infection: Gill filaments (histozoic).
- Materials deposited: Hapantotype no. 21656 (specimens in fixatives), Meguro Parasitological Museum, Tokyo, Japan.
- Deposited rDNA sequence: DDBJ/EMBL/GenBank accession no. LC544125.
- Etymology: The species name refers to the former genus name of the host fish.

### 3.2. Henneguya pungitii Achmerov, 1953 (Myxosporea: Bivalvulida: Myxobolidae)

**Figure 4:**
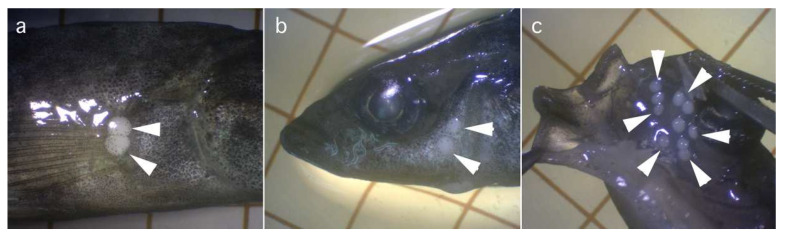
Photomicrographs of plasmodia (arrowheads) of *Henneguya pungitii* at the subcutis of the external surface (**a**,**b**) and oral submucosa (**c**) of *Pungitius sinensis* under a dissection microscope. One grid = 5 mm.

**Figure 5:**
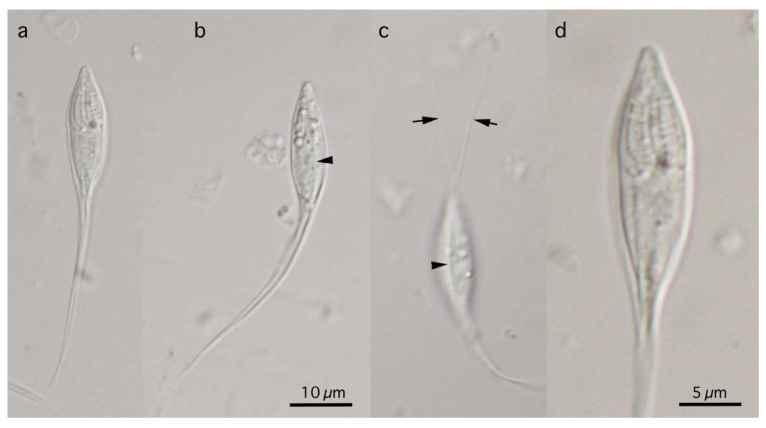
Photomicrographs of myxospores of *Henneguya pungitii*. Frontal view nearly at a sagittal plane (**a**,**d**), and sutural view nearly at surface (**b**,**c**). Photographs (**a**–**c**) at the same magnification with scale bar in (**b**). Photograph (**d**) is a twice-magnified view of the spore body shown in a photograph (**a**). Suture lines (arrowhead) and polar tubules (arrow) are shown.

(Figure 1b,c, Figure 4 and Figure 5)

Oval whitish cysts, measuring 1.1–1.5 mm in diameter, were found in the subcutis of the anterior body and oral submucosa of an Amur stickleback, *Pungitius sinensis* (Figure 4). From the subcutis of the ninespine stickleback, *Pungitius pungitius* (L., 1758), and Sakhalin stickleback, *Pungitius tymensis* (Nikolskii, 1889), in the Russian Far East (Rivers flowing to the Sea of Okhotsk on Kamchatka Peninsula and Sakhalin Island), similar cysts containing myxospores of *Henneguya pungitii* Achmerov, 1953, were recorded [22,35]. Recently, Dorovskikh [39] recorded myxospores of the same species from plasmodial cysts localized in the gills and liver of the ninespine sticklebacks from rivers on the Kolguyey Island, near the Kanin Peninsula, in northwestern Russia. Myxospores of *H. pungitii* recovered from the subcutis and submucosa, or from the gills and liver of *Pungitius* spp., distributed in northeastern and northwestern Russia, respectively, are relatively closer in morphology with each other, but apparently different in size (Table 2). For further research in the future, *H. pungitii* myxospores recovered from the cutaneous subcutis and oral submucosa of Amur Stickleback are described in detail here, along with its molecular–genetic characterization.

**Table 2:** *Henneguya* spp. recorded from freshwater fish, resembling *H. pungitii* in spore shape and dimension ^a^.

Species	Host Fish	Location in Host	Locality	TSL	SBL	SBW	SBT	PCL	PCW	CP	NT	Cyst Size	Reference
*H. pungitii* Achmerov, 1953	*Pungitius sinensis*	Subcutis of External Surface and Oral Submucosa	Japan (Hokkaido)	47.5–56.1 (49.6)	19.3–22.9 (21.0)	6.3–7.6 (6.8)	4.8–6.0 (5.6)	7.8–9.5 (8.4)	1.8–2.5 (2.1)	28.2–33.2 (28.6)	7–8	1.1–1.5 mm in Diameter, Oval	Present Study
*H. pungitii* Achmerov, 1953	*Pungitius pungitius*	Subcutis	Basins of Kamchatka and Paratunka Rivers (Russia)	—	13.0–17.0	4.5–6.0	—	6.5–8.0	1.8–2.0	18.0–20.0	—	1–2 mm in diameter, round	[22]
*H. pungitii* Achmerov, 1953	*Pungitius tymensis* (Nikolskii, 1889)	Subcutis	Sakhalin Island (Russia)	52.9–62.2 (58.2)	16.7–20.6 (18.4)	4.7–5.7 (5.2)	—	6.7–9.0 (8.2)	2.1–2.6 (2.3)	36.2–45.5 (39.8)	—	—	[35]
*H. pungitii* sensu Dorovskikh 2022	*Pungitius pungitius*	Gills	Kolguyev Island, near Kanin Peninsula (Russia)	35.5–40.2 (37.5)	14.7–16.7 (16.1)	5.4–6.0 (5.8)	4.0	6.7–7.4 (7.1)	1.3–2.0 (1.6)	20.8–23.5 (21.4)	—	—	[39]
*H. pungitii* sensu Dorovskikh 2022	*Pungitius pungitius*	Liver	Kolguyev Island, near Kanin Peninsula (Russia)	27.5–32.8 (30.0)	11.4–13.4 (12.7)	6.1–7.7 (6.8)	5.4–6.7 (5.7)	5.4–7.4 (6.6)	2.0–2.7 (2.5)	14.7–19.4 (17.1)	—	—	[39]
*H. alexeevi* Shulman, 1962	*Hypophthalmichthys molitrix* (Valenciennes, 1844); *Perccottus glenii* Dybowski, 1877 (syn. *Percottus glehni* Dybowski, 1877)	Gills, ovary	Amur basin	40.8–52.8 (48.0)	16.8–19.2 (18.2)	5.0–7.2 (6.0)	4.0–5.2 (4.8)	7.8–10.8 (8.9)	1.8–2.2 (1.9)	24.0–33.6 (29.8)	6–7	Cyst: round to oval	[23]
*H. sinensis* Chen et Hsieh, 1960	*Channa argus* (Cantor, 1842); *Clarias batrachus* (L., 1758); *Misgurnus anguillicaudatus* (Cantor, 1842)	Gills, external surface, fin, oral cavity, intestine, swimming bladder	China	31.8–52.2 (42.7)	13.8–16.2 (15.1)	4.8–6.0 (5.4)	3.6–4.2 (3.7)	7.2–8.4 (7.9)	1.4–2.4 (1.9)	18.0–36.0 (27.6)	7–8	0.04–0.28 mm in diameter, round	[23]
*H. rhinogobii* Li et Nie in Li et al., 1973	*Rhinogobius giurinus* (Rutter, 1897); *Rhodeus ocellatus* (Kner, 1866)	Gills, intestine	China, Japan (Gifu)	34.7–59.3 (47.2)	15.4–19.3 (16.9)	4.6–6.2 (5.5)	4.2–5.4 (4.3)	6.9–9.2 (7.9)	1.5–2.0 (1.9)	19.3–40.0 (30.3)	9–10	0.42–1.20 mm by 0.29–0.52 mm, oval	[23]
*H. pseudorhinogobii* Kageyama et al., 2009	*Rhinogobius kurodai* (Tanaka, 1908) (syn. *Rhinogobius* sp. OR)	Gills	Japan (Gifu)	39.5–60.7 (50.7)	14.2–17.8 (15.8)	4.7–5.8 (3.5)	4.5–5.4 (4.8)	5.9–7.6 (6.5)	1.1–1.7 (1.4)	25.3–42.9 (34.9)	8	0.05–0.25 mm in diameter	[40]
*H. pilosa* Azevedo et Matos, 2003	*Serrasalmus altuvei* Ramírez, 1965	Gills	Brazil	52.3–56.0 (54.2)	20.0–23.1 (21.1)	5.5–6.3 (5.9)	1.9–2.6 (2.2)	7.1–7.6 (7.4)	1.0–1.3 (1.2)	30.5–34.9 (31.1)	11–12	max. 0.2 mm in diameter, round to ellipsoidal	[41]

^a^ Abbreviation: TSL, total spore length; SBL, spore body length; SBW, spore body width; SBT spore body thickness; PCL, polar capsule length; PCW, polar capsule width; CP, length of caudal processes; NT, number of coil turns (polar tubules). All measurements are expressed in micrometers (µm) unless otherwise stated. Ranges are presented, with the means in parentheses.

#### 3.2.1. Description

Bivalvular myxospores with elongated spindle-shaped myxospore bodies and long caudal processes of individual valves. Myxospore bodies measuring 19.3–22.9 (21.0) in length, 6.3–7.6 (6.8) in width, and 4.8–6.0 (6.8) in thickness (n = 20). Two almost equal PCs, elongated pyriform, 7.8–9.5 (8.4) in length and 1.8–2.5 (2.1) in width. Binucleated sporoplasm in the remaining space. Turns of polar tubules 7–8. Caudal processes long and fine, measuring 28.2–33.2 (28.6) in length, and total myxospore length 47.5–56.1 (49.6). Valvular surface smooth.

#### 3.2.2. Molecular-Genetic Characterization

A newly obtained, almost complete SSU rDNA nucleotide sequence was 2060 bp in length (accession no. LC544126) and showed the highest identities (91.7% [1741/1899] with 26 indels, or 91.6% [1878/2050] with 16 indels) with sequences from an unidentified *Henneguya* sp. and the aurantiactinomyxon KAB-2001 isolate (accession nos. U13826 and AF378356, respectively).

#### 3.2.3. Remarks

Bivalvular myxospores of this species had two caudal processes which continued from each valve, and two elongated PCs were placed on the sutural plane, consistent with the definition of the genus *Henneguya* according to Lom and Dyková [6]. It is important to note that *H. pungitii* Achmerov, 1953, is characterized by fusiform myxospore bodies tapering to a point at both ends, although *Henneguya* spp. with myxospores having ellipsoid and rounded myxospore bodies in the frontal view are apparently predominant [12,18,20]. *Henneguya* spp. with myxospores exhibiting similar morphological characteristics to those of *H. pungitii* are selected in Table 2. *Henneguya pungitii* sensu Dorovskikh, 2022 [39], and the other five species have different organ preferences, e.g., the gills or visceral organs such as the liver, ovary, intestine, or swimming bladder and their myxospores are characterized by shorter (*H. pungitii* sensu Dorovskikh, 2022, *H. sinensis*, *H. rhinogobii*, and *H. pseudorhinogobii*) and/or thinner myxospore bodies (*H. alexeevi*, *H. sinensis*, *H. rhinogobii*, *H. pseudorhinogobii,* and *H. pilosa*), smaller PCs (*H. pseudorhinogobii*, and *H. pilosa*), and/or more turns of polar tubules (*H. rhinogobii*, and *H. pilosa*), as shown in Table 2.

Availability of the SSU rDNA nucleotide data of aforementioned *Henneguya* spp. with fusiform myxospore bodies (Table 2) is currently limited to three species (the current isolate of *H. pungitii, H. rhinogobii*, and *H. pseudorhinogobii*), and the latter two species show relatively high identity of the SSU rDNA (95.89% [1843/1922] with 10 indels over a 1930 bp length between accession nos. AB447993 and AB447994) with each other, whereas the SSU rDNA of the current *H. pungitii* isolate (accession no. LC544126) shows relatively low identities with those of *H. rhinogobii* and *H. pseudorhinogobii* (82.84% [1569/1894] with 61 indels over a 1958 bp length, and 87.58% [1664/1900] with 53 indels over a 1958 bp length, respectively). The phylogenetic relationships between *H. pungitii* from the subcutis of *Pungitius* spp., distributed in the Russian Far East, and *H. pungitii* sensu Dorovskikh 2022 from the gills and/or liver of *Prungitiu pungitius,* distributed in the northwestern Russia (Kolguyev Island, near Kanin Peninsula), should be elucidated in future research, since these two species show distinct tissue preferences and somewhat different myxospore morphology regardless of their identical host preference [22,35,39].

#### 3.2.4. Taxonomic Summary of the Present Isolate

- Host: *Pungitius sinensis*, Amur stickleback (Actinopterygii: Gasterosteiformes: Gasterosteidae).
- Locality: Collected in Aqua Totto Gifu, Gifu Prefecture, Japan, following its relocation from a river running through Akkeshi-cho Town, Hokkaido Prefecture, Japan. Circumstantially, the infection might originate from the water in the natural habitat, and not in the aquarium.
- Site of infection: Subcutis of external surface and oral submucosa (histozoic).
- Materials deposited: Hapantotype no. 21657 (specimens in fixatives), Meguro Parasitological Museum, Tokyo, Japan.
- Deposited rDNA sequence: DDBJ/EMBL/GenBank accession no. LC544126.

### 3.3. Myxidium salvelini Konovalov et Shulman, 1966 (Myxosporea: Bivalvulida: Myxidiidae)

**Figure 6:**
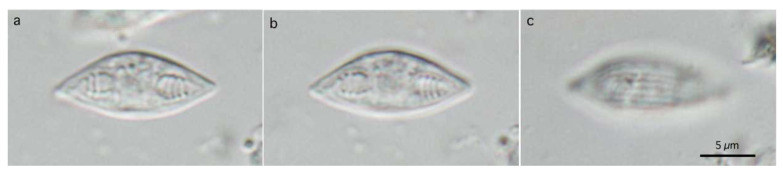
Photomicrographs of myxospores of *Myxidium salvelini*. Frontal view nearly at midline of sagittal plane (**a**,**b**), and spore surface (**c**) at the same magnification. Scale bar is shown in (**c**).

(Figure 1d and Figure 6)

#### 3.3.1. Morphological Characterization

Numerous free myxospores of *M. salvelini* Konovalov et Shulman, 1966, were found in the contents of the urinary bladder from the red spotted masu trout, *Oncorhynchus masou*, while coelozoic plasmodia were never found in the contents or wall of the organ. Bivalvular myxospores were fusiform (Figure 1d and Figure 6), or slightly more arcuate on one side, in the frontal view, measuring 13.6–16.7 (15.0) by 4.9–6.6 (5.7) (n = 20). Fusiform myxo-spores tapered to pointed ends at an angle 60°, and had fine striations (5–7 in number) on the surface of each valve. Two pyriform PCs, measuring 4.0–5.2 (4.4) in length and 2.1–2.8 (2.5) in width, were oriented towards the pointed ends of spores. Binucleated sporo-plasms were located in the middle parts of spores between PCs. Polar filaments formed 4–5 turns in PCs.

#### 3.3.2. Molecular-Genetic Characterization

A newly obtained, almost complete SSU rDNA nucleotide sequence was 2059 bp in length (accession no. LC544127), and showed the highest identity (95.02% [1812/1907] with 9 indels) with *Sphaerospora onchorhynchus* (accession no. AF201373).

#### 3.3.3. Remarks

Myxospores of this myxosporean species were fusiform with pointed ends, and were classified in the genus *Myxidium* according to Lom and Dyková [6]. A synopsis of the *Myxidium* spp. by Eiras et al. [14] included 232 nominal species, of which a majority parasitize the gall bladder as coelozoic myxosporeans, whereas only a small proportion (12.5%) parasitize the urinary system, including the kidney, ureter, and urinary bladder. Similarly, among 19 *Myxidium* spp. recorded in Japan (nine species from freshwater fish, and 12 species from marine fish; see Supplementary Table S1), only *M. lentiforme* (Fujita, 1927) Fujita, 1929 (syn. *M. fusiforme* Fujita, 1927), and *M. uchiyamae* Fujita, 1927, parasitize the urinary system, i.e., kidney, of the Japanese eel (*Anguilla japonica* Temminck et Schlegel, 1846) [42]. These species have morphologically distinct myxospores from those of the present new species (Table 3). Among *Myxidium* spp. recorded in the urinary bladder of freshwater fish worldwide, *M. rimskykorsakowi* Shulman, 1962, and *M. salvelini* have myxospores similar in morphology to those of the current isolate, but the myxospores of the former species are shorter and wider than those of the current isolate, with pointed ends at an angle of 70°–80° and rounded PCs [22]. In contrast, *M. salvelini* recorded from the urinary bladder and ureters of salmonids from the river basin of Kamchatka [22] and the current isolate from the masu salmon in the central Japan (Gifu, Honshu Island of Japan) are coincident in morphology with each other. In Hokkaido, Japan, *M. oncorhynchi* Fujita, 1923, was isolated from the gall bladder of the cherry salmon, *Oncorhynchus masou masou* (Brevoort, 1856) [43]. *Mixidium oncorhynchi* has myxospores with oval to globular PCs, differentiated from *M. salvelini* reported in the present study. No myxosporean species showed highly identical SSU rDNA sequences to that of the current *M. salvelini* isolate (accession no. LC544127). Exclusively, *Sphaerospora onchorhynchus* (accession no. AF201373) showed a high nucleotide identity with a limited number of indels (95.02% [1812/1907] with 9 indels).

#### 3.3.4. Taxonomic Summary of the Present Isolate

- Host: *Oncorhynchus masou*, masu salmon (syn. *Oncorhynchus masou ishikawae*, satsukimasu salmon, or red spotted masu trout) (Actinopterygii: Salmoniformes: Salmonidae).
- Locality: Collected in Aqua Totto Gifu, Gifu Prefecture, Japan, following its relocation from the Nagaragawa River running through Gifu Prefecture, Japan. Circumstantially, the infection might originate from the water in the natural habitat, and not in the aquarium.
- Site of infection: Urinary bladder (coelozoic).
- Materials deposited: Hapantotype no. 21658 (specimens in fixatives), Meguro Parasitological Museum, Tokyo, Japan.
- Deposited rDNA sequence: DDBJ/EMBL/GenBank accession no. LC544127.

### 3.4. Phylogenetic Analysis

**Figure 7:**
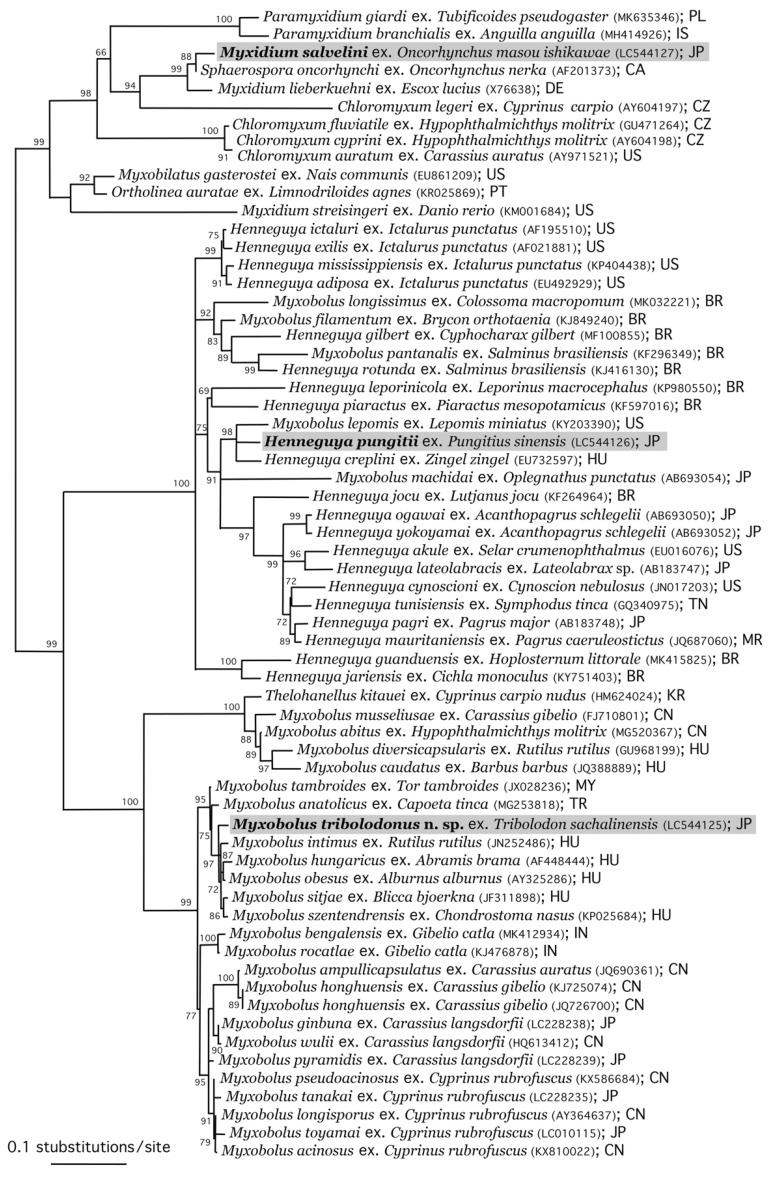
Maximum likelihood phylogenetic tree based on the SSU rDNA sequence of representative myxosporeans of the Bivalvulida. Species names are followed by host fish species, DDBJ/EMBL/GenBank accession numbers in parentheses, and country names as the collection localities. Abbreviations of country names: BR, Brazil; CA, Canada; CN, China; CZ, Czech Republic; DE, Germany; HU, Hungary; IN, India; IS, Iceland; JP, Japan; MR, Mauritania; KR, Korea; MY, Malaysia; PL, Poland; PT, Portugal; TN, Tunisia; and US, United States. Sequences newly obtained in this study are marked with gray background.

The phylogenetic relationships of the three new isolates described above with other myxospoean species are shown in Figure 7. *Myxobolus tribolodonus* sp. n., parasitizing the gills of the rosyface dace, formed a highly supported clade with *M. intimus* Zaika, 1965, from the gill lamellae of the common roach *Rutilus rutilus* (L., 1758); *M. hungaricus* Jaczó, 1940, from the gill filaments of *Abramis brama; M. obesus* from the gills of *Alburmus alburmus*; *M. sitjae* Cech et al., 2012, from the gill filaments of *Blicca bjoerkna*; and *M. szentendrensis* from the gills of the common nase *Chondrostoma nasus* (L., 1758). All these myxobolid species with pyriform myxospores parasitize the gill filaments of cyprinid fishes of the subfamily Leuciscinae, distributed in the northern Far East (*M. tribolodonus* sp. n.) and Europe (the other five species). This clade is the sister group to the clades of myxobolid species parasitizing the gills of cyprinid fishes of the subfamilies Cyprininae (China and Japan) and Labeoninae (India).

*Henneguya pungitii*, parasitizing the external subcutis and oral submucosa of the Amur stickleback, forms a clade with *Henneguya creplini* (Gurley, 1894) Labbé, 1899, from the gills of the zingel *Zingel zingel* (L., 1758) in Europe, and with *Myxobolus lepomis* Rosser et al., 2017, from the gills of the redspotted sunfish *Lepomis miniatus* (Jordan, 1877) and the dollar sunfish *Lepomis marginatus* (Holbrook, 1855) in Texas, USA. The SSU rDNA sequences of these three species showed 86.14% [1392/1616] to 90.30% [1461/1618] identities and 9–18 indels with each other. This clade is the sister group to the clade of *Henneguya* spp. from marine fish.

*Myxidium salvelini*, parasitizing the urinary bladder of the satsukimasu salmon, formed a highly supported clade with *Sphaerospora onchorhynchus* Kent et al., 1993, from the kidney of the sockeye salmon *Oncorhynchus nerka* (Walbaum, 1792) in the North Pacific Ocean, and *Myxidium lieberkuehni* Bütschli, 1882, from the kidney of the northern pike *Esox lucius* L., 1758, in the north parts of the Northern Hemisphere. This clade is rather isolated from other myxosporeans (Figure 7; see also Figure 4 of Sekiya et al. [25]).

## 4. Discussion

Classical taxonomy of Myxozoa based on morphological criteria uses phenotypical characteristics of myxospores such as the number of SVs and PCs, arrangement of the PCs, and SV ornamentation, together with host specificity, site preference of the plasmodium, and geographical distribution [6,8]. A high degree of phenotypical plasticity of myxospores and multiple examples of convergent evolution often render the specific identification difficult [37,49,50,51,52,53,54,55,56,57]. Currently, phylogenetic analyses based on nucleotide sequences of SSU rDNA and/or other genes, e.g., the internal transcribed spacer region 1, the elongation factor 2 gene, mitochondrial cytochrome *c* oxidase (*cox-1*), and mitochondrial small and large subunit ribosomal RNA genes, have become ineluctable techniques for reliable species differentiation [5,21,58,59]. An apparent obstruction for the current taxonomic approach is the unavailability of sufficient molecular–genetic data of myxosporeans, particularly those of the species recorded from wild fish.

In the present study, three new myxosporean isolates from Japan, i.e., *Myxobolus tribolodonus* sp. n. (syn. *M. marinus* sensu Aseeva, 2000; and *M. marinus* sensu Sokolov et Frolova, 2015 from the gills of *Pseudaspius* spp. in the rivers on the Sakhalin Island and Maritime Province (Primorsky Kray) of Russian Far East), *Henneguya pungitii*, and *Mixidium salvelini*, could be differentiated based on the classical taxonomic criteria. All of these species from freshwater fishes in Japan are new distribution records, but their host species have been recorded for each species from the Russian Far East [24,35]. Concurrent molecular–genetic characterization of these new isolates of known species could make it possible to perform reliable identification of the species isolated from the field and clarify their phylogenetic relationships with closely-related species in morphology and/or other biological traits (host specificity, site preference, geographical distribution, etc.). As discussed above, Russian researchers [34,35] identified *Myxobolus* sp. from the gills of *Pseudaspius* spp. (Cyprinidae) in the Russian Far East as *M. marinus* Dogiel, 1948, which had been synonymized by Shulman [22] to *Myxobolus macrocapsularis,* parasitizing the gills and other visceral organs of a variety of cyprids. Morphologically, these two isolates, i.e., *M. marinus* sensu Aseeva, 2000 and *M. marinus* sensu Sokolov et Frolova, 2015, are identical to the new isolate from the gills of *Pseudaspius sachalinensis* in Hokkaido, Japan, although the myxospores of the former isolates are marginally larger than those of the Japanese isolate. It is interesting to explore phylogenetic relationships of species collected from geographically separated host populations (Russian Far East and Hokkaido Island of Japan).

*Henneguya pungitii* isolates from the subcutis of *Prungitius* spp., distributed in the Russian Far East and Hokkaido Island of Japan, show highly identical morphology of myxospores (Table 3). On the other hand, *H. pungitii* from *Pungitius pungitius* in the rivers on Kolguyev Island, near Kanin Peninsula (Russia), which was reported recently by Dorovskikh [39], showed site preferences to the gills and liver, and its myxospores were apparently smaller than the isolates from the subcutis of the same host distributed in the Russian Far East and Hokkaido. *Prungitius* spp. are known to have multiple evolutionarily, geographically, and ecologically separated populations [60]. It is interesting to reveal coevolutional relationships of myxosporeans with their hosts using phenotypically and geographically separated populations of *H. pungitii*. In addition to the possible coevolutionary speciation, sympatric speciation of myxosporeans through occupation of distinct microhabitats within a single host species has also been suggested [53,61].

Currently, it is generally recognized that there is no phylogenetically consistent pattern congruent with host family, tissue tropism, or myxospore morphology [53,61]. At individual clade levels, however, apparent clustering of myxosporeans originating from the same order/family of the host fish has been demonstrated by many studies [3,62,63,64]. Moreover, polyphyly or paraphyly of multiple myxosporean genera such as *Myxobolus*, *Henneguya*, *Sphaerospora*, *Myxidium*, *Zschokkella*, and *Chloromyxum* have been demonstrated [8,25,58,59,61,65,66]. These phylogenetic relationships of taxa classified in different genera are reflected in Figure 7 in the present study.

## 5. Conclusions

The majority of myxosporean species of the genera *Myxobolus*, *Henneguya*, and *Myxidium* from natural freshwater fish in Japan (Supplementary Table S1) remain to be molecular-genetically characterized. Synchronized efforts to accumulate at least SSU rDNA sequences of known or unknown species worldwide will be invaluable, not only for reliable species identification, but also for further understanding of evolutional history and global dispersion of different myxosporean species.

## Figures and Tables

**Table 3:** *Myxidium* spp. resembling *M. salvelini* in spore shape and dimension ^a^.

Species	Host Fish	Location in Host	Locality	SL	SW	ST	PCL	PCW	NT	NVS	Reference
*M. salvelini*	*Oncorhynchus masou* (syn. *Oncorhynchus masou ishikawae*)	Urinary Bladder	Japan (Gifu)	13.6–16.7 (15.0)	4.9–6.6 (5.7)	—	4.0–5.2 (4.4)	2.1–2.8 (2.5)	4–5	5–7	Present Study
*M. salvelini*	*Salvelinus alpinus* (L., 1758); *Salvelinus leucomaenis* (Pallas, 1814); *Oncorhynchus mykiss* (Walbaum, 1792) (syn. *Salmo mykiss* Walbaum, 1792)	Urinary bladder and ureters	Basin of Kamchatka River	12.0–16.0	5.2–6.4	4.6–6.0	4.6–5.3	2.7–3.5	—	ca. 6	[22]
*M. americanum* Kudo, 1920	*Apalone spinifera* (Lesueur, 1827) (syn. *Trionyx spinifera* Lesueur, 1827)	Urinary tubules (Kidney)	USA (Illinois)	15–16	5.5–6	—	4	3.5	3	8–10	[44]
*M. carinae* Alvarez-Pellitero et al., 1983	*Luciobarbus bocagei* (Steindachner, 1864) (syn. *Barbus bocagei* Steindachner, 1864)	Gall bladder	Spain	12.0–14.8 (13.4)	5.0–7.0 (6.0)	5.5–8.0 (6.7)	3.0–5.3 (4.5)	2.8–4.6 (3.7)	4–5	10–11/8–9	[45]
*M. clariae* Landsberg, 1987	*Clarias gariepinus* Burchell, 1822 (syn. *Clarias lazera* Valenciennes, 1840)	Gall bladder	Israel	13.4–15.1 (14.3)	4.5–6.0 (5.3)	—	3.6–4.8 (4.2)	3.0–3.8 (3.3)	5–6	7–9	[46]
*M. oncorhynchi*	*Oncorhynchus masou* (syn. *Oncorhynchus masou masou*)	Gall bladder	Japan (Hokkaido)	11–12	5–8	3–5	3–4 in diameter (oval to globular)		—	ca. 5	[43]
*M. rimskykorsakowi*	*Perccottus glenii* Dybowski, 1877	Urinary bladder	Amur basin	12.0–13.0	6.5–7.0	5.0	4.5	3.0–3.5	—	ca. 5	[22]
*M. lentiforme* (syn. *Myxidium fusiformis* Fujita, 1927)	*Anguilla japonica*	Kidney	Japan (Shiga)	19	5	4	4	—	—	—	[42,47]
*M. sinilabi* Feng et Xiao, 1998	*Decorus rendahli* (Kimura, 1934) (syn. *Sinilabeo rendahli rendhali* (Kimura, 1934); *Pseudogyrinocheilus prochilus* (Sauvage et Dabry de Thiersant, 1874) (syn. *Semilabeo prochilus* (Sauvage et Dabry de Thiersant, 1874)	Gall bladder	China	15.5–18.0 (16.0)	5.0–7.0 (6.0)	—	3.5–5.5 (5.0)	3.0–4.1 (4.0)	4	7–10	[23]
*M. striatusi* Sarkar, 1982	*Channa striata* (Bloch, 1793) (syn. *Ophicephalus striatus* Bloch, 1793)	Gall bladder	India	11.1–18.7 (14.5)	4.7–7.0 (5.6)	—	3.7–5.6 (4.5)	2.8–3.7 (3.0)	—	—	[48]
*M. truttae* Lèger, 1930	*Salmo trutta* L., 1758 (syn. *Salmo trutta fario* L., 1758)	Gall bladder	France	11–12	7–7.3	—	3.7 in diamer in diameter (oval)		—	—	[14]
*M. uchiyamae*	*Anguilla japonica*	Kidney	Japan (Shiga)	13.5	8	6	6.5	—	—	—	[42]

^a^ Abbreviation: SL, spore length; SW, spore width; ST spore thickness; PCL, polar capsule length; PCW, polar capsule width; NT, number of coil turns (polar tubles); and NVS, number of vulvar striations. All measurements are expressed in micrometers (µm) unless otherwise stated. Ranges with the means in parentheses are presented. For *M. flavus*, means and standard variation are shown in parentheses.

## Data Availability

Myxosporean specimens collected in this study are deposited in the Meguro Parasitological Museum, Tokyo, Japan, under collection nos. 21656–21658. The nucleotide sequence obtained in this study is available from the DDBJ/EMBL/GenBank databases under accession no. LC544125–LC544127. The data that support the findings of this study are available from the corresponding author upon reasonable request.

## Supplementary Materials

The following are available online at https://www.mdpi.com/article/10.3390/life14080974/s1, Supplementary Table S1: Myxosporean species of the genera *Myxobolus*, *Henneguya,* and *Myxidium*, recorded in Japan; Supplementary Table S2: Fish specimens examined in our survey of myxosporeans in freshwater fish provided by Aquatotto Gifu, Japan; and Supplementary Table S3: *Myxobolus* spp. parasitizing the gills of freshwater fish, with myxospore length (SL) ranging between 7 µm and 10 µm and almost equal-sized polar capsules, like *M. tribolodonus* sp. n. All references cited in the supplementary materials [9,22,23,28,29,40,42,43,44,47,67,68,69,70,71,72,73,74,75,76,77,78,79,80,81,82,83,84,85,86,87,88,89,90,91,92,93,94,95,96,97,98,99,100,101,102,103,104,105,106,107,108,109,110,111,112,113,114,115,116,117,118] are included in the reference list below.
