# Supplementary material for: Morphological and Molecular Characterization of Three Myxosporean Species of the Genera Myxobolus, Henneguya, and Myxidium (Cnidaria: Myxozoa) Infecting Freshwater Fish, Isolated for the First Time in Japan
Source: Life (Basel). 2024 Aug 2;14(8):974. doi: 10.3390/life14080974 (PMC11355902; doi:10.3390/life14080974)
Supplement: Supplementary file 1 [file life-14-00974-s001.zip › life-3065167-supplementary.pdf]

**Suppl. Table S1.** Myxosporean species of the genera *Myxobolus*, *Henneguya* and *Myxidium*, recorded in Japan

| | ***Myxobolus* Bütschli, 1882** | Host fish | Location of parasitism | GenBank accession no. | Reference |
|---|---|---|---|---|---|
| 1 | *M. acheilognathi* (Fujita, 1927) Landsberg et Lom, 1991<br>syn. *Leptotheca acheilognathi* Fujita, 1927; *Myxosoma acheilognathi* (Fujita, 1927) Kudo, 1933 | *Acheilognathus lanceolatus* | Subcutis | — | [42, 67, 68] |
| 2 | *M. acutus* (Fujita, 1912) Landsberg et Lom, 1991<br>syn. *Sphaerospora acuta* Fujita, 1912; *Myxosoma acutum* (Fujita, 1912) Kudo, 1920 | *Carassius gibelio* | Gill | — | [44, 68, 69] |
| 3 | *M. arcticus* Pugachev et Khokhlov, 1979 | *Oncorhynchus masou* | Central nervous system | AF085176; AB353128–AB353130; AB469990–AB469993; HQ113228 | [70–73] |
| 4 | *M. artus* Akhmerov, 1960 | *Cyprinus carpio* | Muscle | FJ710799 | [74, 75] |
| 5 | *M. concentricus* (Ozaki et Ishizaki, 1941) Landsberg et Lom, 1991 | *Tridentiger obscurus* | Urinary bladder | — | [68, 76] |
| 6 | *M. cultus* Yokoyama et al., 1995 | *Carassius auratus* | Cartilage | AB121146; HQ613409; KY784597–KY784601 | [77] |
| 7 | *M. dermatobius* (Ishii, 1915) Landsberg et Lom, 1991<br>syn. *Lentospora dermatobia* Ishii, 1915; *Myxosoma dermatobium* (Ishii, 1915) Kudo, 1920 | *Anguilla japonica* | Skin | — | [44, 78, 79] |
| 8 | *M. dujardini* (Thélohan, 1895) Landsberg et Lom, 1991<br>syn. *Myxosoma dujardini* Thélohan, 1895 | *Cyprinus carpio* | Gill | DQ439803: DQ439804 | [80] |
| 9 | *M. ellipticus* (Fujita, 1924) Nagasawa et al., 1989<br>syn. *Lentospora elliptica* Fujita, 1924; *Myxosoma ellipticum* (Fujita, 1924) Kudo, 1933 | *Carassius vulgaris* | Kidney | — | [67, 81, 82] |
| 10 | *M. elongatus* Fujita, 1924 | *Carassius carassius* | Kidney | — | [81] |
| 11 | *M. fujitai* (Fujita, 1929) Eiras et al., 2005<br>syn. *Lentospora anguillae* Fujita, 1929; *Myxosoma anguillae* (Fujita, 1929) Kudo, 1933 | *Anguilla japonica* | Fin, skin | — | [9, 47, 67] |
| 12 | *M. gigi* (Fujita, 1927) Shulman, 1962<br>syn. *Lentospora gigi* Fujita, 1927; *Myxosoma gigi* (Fujita, 1927) Kudo, 1933 | *Fulvidraco nudiceps* | Kidney | — | [22, 42, 67] |
| 13 | *M. ginbuna* Kato et al., 2017 | *Carassius langsdorfii* | Gill | LC228238 | [29] |
| 14 | *M. gnathopogonae* (Inoue et Hoshina, 1983) Landsberg et Lom, 1991<br>syn. *Myxosoma gnathopogonae* Inoue et Hoshina, 1983 | Gnathopogon elongatus caerulescens | Skin | — | [68, 83] |
| 15 | *M. hoshinai* (Hoshina, 1953) Landsberg et Lom, 1991 | *Cyprinus carpio* | Skin | — | [68, 79] |

| | | | | | |
|---|---|---|---|---|---|
| | syn. *Myxobolus dermatobius* Hoshina, 1953 | | | | |
| 16 | *M. kawabatae* (Fujita, 1927) Shulman, 1962<br>syn. *Lentospora kawabatae* Fujita, 1927; *Myxoxoma kawabatai* (Fujita, 1927) Kudo, 1933 | *Fulvidraco nudiceps* | Kidney | — | [22, 42, 67] |
| 17 | *M. koi* Kudo, 1920 | *Cyprinus carpio* | Gill | FJ710800; FJ841887; KT240127; KJ725077; MH196560 | [44, 84–86] |
| 18 | *M. leucogobianus* (Fujita, 1927) Landsberg et Lom, 1991<br>syn. *Lentospora leucogobiana* Fujita, 1927; *Myxosoma leucogobianum* (Fujita, 1927) Kudo, 1933 | *Pseudogobius esocinus* | Kidney | — | [42, 68] |
| 19 | *M. luciogobii* (Ishizaki, 1957) Landsberg et Lom, 1991<br>syn. *Myxosoma luciogobii* Ishizaki, 1957 | *Luciogobius guttatus* | Urinary bladder | — | [68, 87] |
| 20 | *M. marumotoi* Li et Sato, 2014 | *Odontobutis obscura* | Muscle | AB873006 | [88] |
| 21 | *M. miyairii* Kudo, 1920 | *Parasilurus asotus* | Intestine | KT001495 | [44] |
| 22 | *M. murakamii* Urawa et al., 2009 | *Onchorhynchus masou masou*; *Onchorhynchus masou ishikawae* | Lateral line nerve | AB469984 | [89] |
| 23 | *M. nagaraensis* Yokoyama et al., 2007 | *Rhinogobius kurodai* | Body cavity (kidney); caudal peduncle | AB274267 | [90] |
| 24 | *M. neurobius* Schuberg et Schröder, 1905 | *Onchorhynchus masou*; *Onchorhynchus rhodurus* | Spinal cord | AF085180; AB469986; AB469987 | [70, 71, 89] |
| 25 | *M. pseudoacinosus* (Kato et al., 2017) Guo et al., 2018<br>syn. *Myxobolus paratoyamai* Kato et al., 2017 | *Cyprinus carpio* | Gill | LC228237; KX586684; KX810019; KX810020 | [29, 91] |
| 26 | *M. pseudorasbore* (Hoshina, 1952) Landsberg et Lom, 1991<br>syn. *Myxosoma pseudorasbore* Hoshina, 1952 | *Pseudorasbora parva* | Muscle | — | [68, 85] |
| 27 | *M. pyramidis* Chen in Chen et Ma, 1998 | *Carassius auratus*; *Carassius langsdorfii* | Gill | HQ613411; LC228239 | [29] |
| 28 | *M. sacchalinensis* (Fujita, 1924) Landsberg et Lom, 1991<br>syn. *Lentospora sacchalinensis* Fujita, 1924; *Myxosoma sacchalinensis* (Fujita, 1924) Kudo, 1933 | *Carassius gibelio* | Kidney | — | [68, 81] |
| 29 | *M. salmonis* (Hoshina, 1949) Landsberg et Lom, 1991<br>syn. *Myxosoma salmonis* Hoshina, 1949 | *Oncorhynchus keta* | Scale | — | [68, 92] |
| 30 | *M. sphaericus* (Fujita, 1924) Landsberg et Lom, 1991<br>syn. *Lentospora sphaerica* Fujita, 1924; *Myxosoma sphaericum* (Fujita, 1924) Kudo, 1933 | *Carassius gibelio* | Kidney | — | [68, 81] |
| 31 | *M. taiwanensis* (Fujita, 1924) Landsberg et Lom, 1991<br>syn. *Lentospora taiwanensis* Fujita, 1924; *Myxosoma taiwanensis* (Fujita, 1924) Kudo, 1933 | *Carassius carassius*; *Carassius vulgaris* | Kidney | — | [68, 81] |
| 32 | *M. tanakai* Kato et al., 2017 | *Cyprinus carpio* | Gill | LC228235; LC228236; | [29] |

| | | | | | |
|---|---|---|---|---|---|
| | | | | MH196559; MK552413; MK552414 | |
| 33 | *M. toyamai* Kudo, 1917 | *Cyprinus carpio* | Gill | FJ710802; LC010115; LC010116; | [29, 44, 93, 94] |
| **34** | ***Myxobolus tribolodonus* sp. n.** | ***Pseudaspius sachalinensis* (syn. *Tribolodon sachalinensis*)** | **Gill** | **LC544125** | **Present study** |
| 35 | *M. uniporus* Fujita, 1927 | *Parasilurus asotus* | Kidney | — | [42] |
| 36 | *M. wulii* (Wu et Li, 1986) Landsberg et Lom, 1991 | *Carassius auratus* | Gill, hepatopancreas | EF690300; HQ613412; KP642131; KP642132; KJ725081; MH920541; MH920542; KY784580 | [95] |
| [Marine species] | | | | | |
| 37 | *M. acanthogobii* Hoshina, 1952 syn. *Myxobolus buri* Egusa, 1985 | *Acanthogobius flavimanus*; *Seriola quinqueradiata*; *Scomber japonicus*; *Evynnis japonica*; *Lepidotrigia alata*; *Canthigaster rivulata* | Brain; Periphery of eyes | — | [85, 96–100] |
| 38 | *M. episquamalis* Egusa et al., 1990 | *Mugil cephalus* | Beneath the scales, fins, gill arches | AY129312; JF810537; KC733437; MK329248 | [101, 102] |
| 39 | *M. machidai* Li et al., 2012 | *Oplegnathus punctatus* | Esophageal wall | AB693054 | [28] |
| 40 | *M. spinacurvatura* Maeno et al., 1990 | *Mugil cephalus* | Brain, viscera | AF378341 | [103] |
| 41 | *M. spirosulcatus* Maeno et al. 1995 | *Seriola quinqueradiata* | Bile duct; brain | AB530261–AB530263 | [97, 104] |

| | ***Henneguya* Thélohan, 1892** | Host fish | Location of parasitism | GenBank accession no. | Reference |
|---|---|---|---|---|---|
| 1 | *H. cartilaginis* Yokoyama et al., 2012 | *Oncorhynchus masou masou* | Head cartilage | — | [105] |
| 2 | *H. miyairii* Kudo, 1920 | *Carassius auratus* | Head subcutis | — | [44] |
| 3 | *H. miyazakii* Hoshina, 1952 | *Chenogobius annularis* | Subcutis | — | [85] |
| 4 | *H. mogurndae* Fujita, 1936 | *Mogurnda obscura* | Gill | — | [106] |
| 5 | *H. postexilis* Minchew, 1977 | *Ictalurus punctatus* | Gill | LC781947 | [107] |
| 6 | *H. preintestinalis* Ozaki et Isizaki, 1941 | *Tridentiger obscurus* | Intestine | — | [76] |
| 7 | *H. pseudorhinogobii* Kageyama et al., 2009 | *Rhinogobius* sp. OR | Gill | AB447994–AB447996 | [40] |
| **8** | ***H. pungitii* Achmerov, 1953** | ***Pungitius sinensis*** | **Subcutis, oral submucosa** | **LC544126** | **Present study** |
| 9 | *H. rhinogobii* Li et Nie, 1973 | *Rhinogobius* sp. OR | Gill | AB447992; AB447993 | [40] |
| 10 | *H. tridentigeri* Ozaki et Ishizaki, 1941 | *Tridentiger obscurus*; *Acanthogobius flavimanus* | Skin; gill | — | [76, 85] |
| [Marine species] | | | | | |
| 11 | *H. lateolabracis* Yokoyama et al., 2003 | *Lateolabrax* sp. | Aortic bulb | AB183747 | [108] |
| 12 | *H. miyazakii* Hoshina, 1952 | *Chaenogobius annularis* | Skin | — | [85] |
| 13 | *H. pagri* Yokoyama et al., 2005 | *Pagrus major* | Aortic bulb | AB183748 | [109] |
| 14 | *H. ogawai* Li et al., 2012 | *Acanthopagrus schlegelii* | Intestinal wall | AB693050; AB693051 | [28] |
| 15 | *H. yokoyamai* Li et al., 2012 | *Acanthopagrus schlegelii* | Peritonium | AB693052; AB693053 | [28] |

| *Myxidium Bütschli, 1882* | | Host fish | Location of parasitism | GenBank accession no. | Reference |
|---|---|---|---|---|---|
| 1 | *Myxidium anguillae* (Ishii, 1915) Jayasri et Hoffman, 1982<br>* Hine (1980) suggested that this species is a junior synonym of *Myxidium giardi* Cepède, 1906 | *Anguilla japonica* | Intestine | — | [110–113] |
| 2 | *Myxidium cuneiforme* Fujita, 1924 | *Cyprinus carpio* | Gall bladder | DQ377709; FJ417061; MH497020; MH497021 | [81] |
| 3 | *Myxidium enchelypterygii* Hoshina, 1952<br>* Hine (1980) suggested that this species is a junior synonym of *Myxidium giardi* Cepède, 1906 | *Anguilla japonica* | Fin (dorsal, anal) | — | [85, 111, 113, 114] |
| 4 | *Myxidium ischikauiae* Fujita, 1927 | *Ischikauia steenackeri* | Gall bladder | — | [42] |
| 5 | *Myxidium kagayamai* Kudo, 1920 | *Misgurnus anguillicaudatus* | Gall bladder | — | [44] |
| 6 | *Myxidium lentiforme* (Fujita, 1927) Fujita, 1929<br>syn. *Mixidium fusiformis* Fujita, 1927 | *Anguilla japonica* | Kidney | — | [42, 47] |
| 7 | *Myxidium matsuii* Fujita, 1929 | *Anguilla japonica* | Skin | — | [47, 85] |
| 8 | *Myxidium oncorhynchi* Fujita, 1923 | *Oncorhynchus masou* | Gall bladder | — | [43] |
| 9 | ***Myxidium salvelini* Konovalov et Shulman, 1966** | ***Oncorhynchus masou ishikawae*** | **Urinary bladder** | **LC544127** | **Present study** |
| 10 | *Myxidium uchiyamae* Fujita, 1927 | *Anguilla japonica* | Kidney | — | [42] |
| [Marine species] | | | | | |
| 11 | *Myxidium clidodermatis* Fujita, 1923 | *Clidoderma asperrimum* | Gall bladder | — | [43] |
| 12 | *Myxidium crassum* Fujita, 1923 | *Xystrias grigorjewi* | Gall bladder | — | [43] |
| 13 | *Myxidium eminentis* Ishizaki, 1957 | *Luciogobius guttatus* | Gall bladder | — | [87] |
| 14 | *Myxidium fusiformis* Fujita, 1923 | *Sebastodes flammeus* | Gall bladder | — | [43] |
| 15 | *Myxidium japonicum* Dogiel, 1948 | *Myoxocephalus brandti; Enophrys diceraus; Gymnacanthus herzensteini; Bero elegans; Hemitripterus villosus* | Gall bladder | — | [22] |
| 16 | *Myxidium microcapsulare* Fujita, 1923 | *Atheresthes evermanni* | Gall bladder | — | [43] |
| 17 | *Myxidium microstomi* Fujita, 1923 | *Microstomus stelleri* | Gall bladder | — | [43] |
| 18 | *Myxidium ochotense* Fujita, 1923 | *Gadus macrocephalus* | Gall bladder | — | [43] |
| 19 | *Myxidium oshoroense* Fujita, 1923 | *Paralichthys olivaceus; Hippoglossoides hamiltoni; Lepidopsetta mochigarei; Limanda aspera; Platichythys stellatus; Microstomus stelleri* | Gall bladder | — | [43] |
| 20 | *Myxidium pearcyi* Moser et al., 1976 | *Coelorhynchus macrochir* | Gall bladder | — | [115] |
| 21 | *Myxidium theragrae* Fujita, 1923 | *Theragra chalcogramma* | Gall bladder | — | [43] |
| 22 | *Myxidium tsudae* Fujita, 1923 | *Sebastobolus macrochir* | Gall bladder | — | [43] |

**Additional References (synopsis articles related to genera *Myxobolus*, *Henneguya*, and *Myxidium*): [3–6, 8, 12, 14, 17, 64, 107]**

**Suppl. Table S2.** Fish specimens examined in our survey of myxosporeans in freshwater fish provided by Aquatotto Gifu, Japan

| Order | Family | Species | Number of specimens |
|---|---|---|---|
| Anguilliformes | Anguillidae | *Anguilla japonica* Temminck et Schlegel, 1846 | 1 |
| Cypriniformes | Cyprinidae | *Rhynchocypris lagowskii* (Dybowski, 1869) | 6 |
| Cypriniformes | Cyprinidae | *Tanakia limbata* (Temminck et Schlegel, 1846) | 4 |
| Cypriniformes | Cyprinidae | *Acheilognathus cyanostigma* Jordan et Fowler, 1903 | 1 |
| Cypriniformes | Cyprinidae | *Pseudorasbora pugnax* Kawase et Hosoya, 2015 | 4 |
| Cypriniformes | Cyprinidae | *Tribolodon sachalinensis* (Nikolskii, 1889) | 1 |
| Cypriniformes | Cyprinidae | *Zacco platypus* (Temminck et Schlegel, 1846) | 5 |
| Cypriniformes | Cyprinidae | *Pseudogobio esocinus* (Temminck et Schlegel, 1846) | 2 |
| Cypriniformes | Cyprinidae | *Hemigrammocypris rasborella* Fowler, 1910 | 3 |
| Cypriniformes | Cyprinidae | *Nipponocypris temminckii* (Temminck et Schlegel, 1846) | 2 |
| Cypriniformes | Cyprinidae | *Carassius buergeri langsdorfii* (Temminck et Schlegel, 1846) | 1 |
| Cypriniformes | Cyprinidae | *Rhodeus suigensis* (Mori, 1935) | 1 |
| Cypriniformes | Cyprinidae | *Squalidus japonicus japonicus* Sauvage, 1883 | 2 |
| Cypriniformes | Cyprinidae | *Candidia siebolldii* (Temminck et Schlegel 1846) | 4 |
| Cypriniformes | Cyprinidae | *Hypophthalmichthys molitrix* (Valenciennes, 1844) | 2 |
| Cypriniformes | Cyprinidae | *Tanakia lanceolata* (Temminck et Schlegel, 1846) | 1 |
| Cypriniformes | Cyprinidae | *Acheilognathus tabira tabira* Jordan et Thompson, 1914 | 1 |
| Cypriniformes | Nemacheilidae | *Lefua echigonia* Jordan et Richardson, 1907 | 3 |
| Gasterosteiformes | Gasterosteidae | *Pungitius tymensis* (Nikolskii, 1889) | 5 |
| Gasterosteiformes | Gasterosteidae | *Pungitius* sp. | 4 |
| Gasterosteiformes | Gasterosteidae | *Gasterosteus microcephalus* Girard, 1854 | 12 |
| Osmeriformes | Plecoglossidae | *Plecoglossus altivelis* (Temminck et Schlegel, 1846) | 7 |
| Perciformes | Anabantidae | *Ctenopoma acutirostre* Pellegrin, 1899 | 1 |
| Perciformes | Gobiidae | *Acanthogobius lactipes* (Hilgendorf, 1879) | 5 |
| Perciformes | Gobiidae | *Gymnogobius urotaenia* (Hilgendorf, 1879) | 1 |
| Perciformes | Gobiidae | *Rhinogobius kurodai* (Tanaka, 1908) | 1 |
| Perciformes | Gobiidae | *Tridentiger obscurus* (Temminck et Schlegel, 1845) | 1 |
| Perciformes | Lateolabracidae | *Lateolabrax japonicus* (Cuvier et Valenciennes, 1828) | 1 |
| Pleuronectiformes | Pleuronectidae | *Kareius bicoloratus* (Basilewsky, 1855) | 1 |
| Salmoniformes | Salmonidae | *Oncorhynchus masou masou* (Brevoort, 1856) | 2 |
| Scorpaeniformes | Cottidae | *Cottus kazika* Jordan et Starks, 1904 | 2 |
| Scorpaeniformes | Cottidae | *Cottus pollux* Günther, 1873 | 1 |
| Siluriformes | Amblycipitidae | *Liobagrus reinii* Hilgendorf, 1878 | 2 |
| Siluriformes | Plotosidae | *Plotosus japonicus* Yoshino et Kishimoto, 2008 | 2 |
| 9 orders in total | 13 families in total | 34 species in total | 92 in total |

**Suppl. Table 3.** *Myxobolus* spp. parasitizing the gills of freshwater fish, which had myxospore length (SL) ranging between 7 µm and 10 µm and almost equal-sized polar capsules, like *M. tribolodonus* sp. n.[a]

| Species | Host fish | Location in host | Locality | SL | SW | ST | PCL | PCW | SF | IP | NT | Cyst size | Reference |
|---|---|---|---|---|---|---|---|---|---|---|---|---|---|
| ***M. tribolodonus* sp. n. (syn. *M. marinus* sensu Aseeva, 2000; *M. marinus* sensu Sokolov et Frolova, 2015)** | ***Pseudaspius sachalinensis* (Nikolskii, 1889) (syn. *Tribolodon sachalinensis* (Nikolskii, 1889))** | **Gills** | **Japan (Hokkaido)** | **8.7–9.6 (9.2)** | **6.5–7.5 (7.0)** | **—** | **4.6–6.0 (5.1)** | **1.7–2.1 (1.9)** | **pyriform** | **small** | **4–5** | **max.2.35 by 0.87 mm** | **Present study** |
| *M. acheilognathusi* Ma et Zhao in Chen et Ma, 1998 | *Abbottina rivularis* (Basilewsky, 1855) | Gills | China | 8.8–11.2 (10.3) | 6.4–8.8 (6.8) | 5.4–5.6 (5.5) | 4.0–4.8 (4.4) | 1.6–2.4 (2.2) | pyriform | none | — | 0.16 mm by 0.12 mm | [23] |
| *M. acutus* (Fujita, 1912) Landsberg et Lom, 1991 (syn. *Sphaerospora acuta* Fujita, 1912) | *Carassius gibelio* (Bloch, 1782) | Gills | Japan (Tokyo) | 8–10 | 7–8 | 5–6 | 5 | 4 | ellipsoid | none | — | — | [69] |
| *M. bengalensis* Cakravarty et Basu, 1948 | *Labeo catla* (Hamilton, 1822) (syn. *Catla catla* (Hamilton, 1822); *Gibelion catla* (Hamilton, 1822)) | Gills | India | 8.6–9.4 | 6.4–6.8 | — | 4.3–5.4 | 2.5–3.2 | pyriform | none | 6 | 2.0–4.1 mm in diameter, oval | [48] |
| *M. branchialis* (Markevitsch, 1932) Landsberg et Lom, 1991 (syn. *Lentospora branchialis* Markewitsch, 1932) | *Barbus borysthenicus* Dybowski, 1862 (syn. *Barbus barbus borysthenicus* Dybowski, 1862); *Luciobarbus bocagei* (Steindachner, 1864) | Gills | Ukraine, Hungary | 6.8–8.4<br>7.4–8.5 (8.0) | 5.8–6.4<br>6.3–7.5 (6.8) | 4.0–4.8<br>5.6–5.9 (5.8) | 2.5–3.2<br>3.7–5.2 (4.2) | 1.6–2.0<br>2.2–3.1 (2.7) | ellipsoid | —<br>none | —<br>6 | 0.10–0.15 mm<br>0.060–0.120 mm | [9, 49] |
| *M. brevifilis* (Ma, 1998) Erias et al., 2005 (syn. *Myxosoma chengkiangensis* Ma in Chen et Ma, 1998) | *Folifer brevifilis* (Peters, 1881) (syn. *Tor brevifilis* (Peters, 1881)) | Gills | China | 8.0–10.4 (9.4) | 6.8–7.2 (7.1) | 5.3–6.5 (6.0) | 4.8–5.0 (4.8) | 2.4–2.6 (2.5) | pyriform | small | 6 | 0.45 mm by 0.35 mm | [23] |
| *M. cabedae* (Ghittino, 1962) Landsberg et Lom, 1991 (syn. *Myxosoma cabedae* Ghittino 1962) | *Squalius cephalus* (Linnaeus, 1758) (syn. *Leuciscus cephalus cabeda* Risso, 1827) | Gills | Italy | 8.5–8.7 | 6.8–7.0 | 5.0–5.2 | 5.4–5.6 | — | — | none | — | — | [9] |
| *M. flavus* Carriero et al., 2013 | *Pseudoplatystoma corruscans* (Spix et Agassiz, 1829); *Pseudoplatystoma reticulatum* Eigenmann et Eigenmann, 1889 | Gills | Brazil | (9.2±0.2) | (6.5±0.3) | (4.2±0.2) | (4.5±0.2) | (1.6±0.1) | ellipsoid | none | 4–5 | 1–5 mm in diameter, spherical | [63] |
| *M. funsienensis* (Ma, 1998) Erias et al., 2005 (syn. *Myxosoma fusienenesis* Ma in Chen et Ma, 1998) | *Spinibarbichthys yunnanensis* (Tsü, 1977) (syn. *Spinbarbus denticulatus yunnanensis* (Tsü, 1977)) | Gills | China | 8.8–10.4 (9.5) | 6.8–7.2 (7.0) | 5.6–6.4 (5.9) | 4.2–5.4 (4.7) | 2.0–2.4 (2.3) | pyriform | none | 5 | 0.52-0.75 mm by 0.43-0.50 mm | [23] |
| *M. jianouensis* Wu et al., 1993 | *Leptobotia tientainensis* (Wu, 1930) (syn. *Leptobotia* | Gills | China | 8.8–9.6 (9.3) | 6.6–7.4 (7.2) | 4.4–5.5 (4.7) | 5.2–5.9 (5.6) | 2.6–2.8 (2.6) | ellipsoid | small | — | — | [23] |

| | | | | | | | | | | | | | |
|---|---|---|---|---|---|---|---|---|---|---|---|---|---|
| | *compressicauda* Nichols, 1931) | | | | | | | | | | | | |
| *M. mabianensis* Erias et al., 2005 (syn. *Myxosoma schizothoraxi* Ma in Chen et Ma, 1998) | *Schizothorax prenanti* (Tchang, 1930); *Schizothorax wangchiachii* (Fang, 1936); *Schizothorax meridionalis* Tsao, 1964 | Gills, Kidney | China | 8.8–9.6 (9.4) | 6.4–7.2 (7.0) | 6.4 | 4.0–4.8 (4.5) | 2.4–3.2 (2.7) | pyriform | small | 6–7 | 0.39 mm | [23] |
| *M. nankuensis* Chen in Chen et Ma, 1998 | *Zacco platypus* (Temminck et Schlegel, 1846) | Gills | China | 9.0–10.8 (9.1) | 7.2–8.4 (7.7) | 6.0 | 4.6–4.9 (4.8) | 2.4–2.8 (2.5) | pyriform | none | 6–7 | — | [23] |
| *M. spinibarbus* (Ma, 1998) Erias et al., 2005 (syn. *Myxosoma pyriformis* Ma in Chen et Ma, 1998) | *Spinibarbichthys yunnanensis* (Tsü, 1977) (syn. *Spinbarbus denticulatus yunnanensis* (Tsü, 1977)) | Gills | China | 8.8–9.5 (9.0) | 6.4–7.2 (6.6) | 6.0–6.4 (6.2) | 4.6–5.0 (4.8) | 2.1–2.5 (2.3) | ellipsoid | none | 5 | 0.080 mm by 0.090 mm | [23] |
| *M. tambroides* Székely et al., 2012 | *Tor tambroides* (Bleeker, 1854) | Gills | Malaysia | 8.8–10.6 (9.9) | 6.8–7.9 (7.4) | 7.0–7.9 (7.2) | 5.0–7.0 (5.7) | 2.4–2.9 (2.6) | pyriform | knob-like | 5–6 | — | [116] |
| *M. venkateshi* Senappa et Manohar, 1981 | *Cirrhinus mrigala* (Hamilton, 1822); *Heteropneustes fossilis* (Bloch, 1794) | Gills | India | 9.0–10.0 (9.8) | 7.0–8.0 (7.2) | 5.0 | 5.0–6.0 (5.3) | 2.0 | pyrirom | Small, sharply pointed | 6-7 | — | [117] |
| *M. waleckii* Yukhimenko, 1986 | *Leuciscus waleckii* (Dybowski, 1869) | Gills | Amur basin | 8.4–9.4 | 7.3–8.4 | 5.7–6.3 | 4.2–4.8 | 2.2–3.1 | ellipsoid | small | — | 0.1—0.2 mm | [118] |
| *M. widisuturalis* (Ma et Zhao, 1993) Ma et Zhao, 1994 (syn. *Myxobolus zacconi* Ma et Zhao, 1993) | *Zacco platypus* (Temminck et Schlegel, 1846) | Gills | China | 8.8–9.6 (9.2) | 7.2–8.0 (7.5) | 6.4 | 4.8–6.0 (5.5) | 2.4–3.2 (2.9) | pyrirom | none | — | 0.120 mm by 0.095 mm | [23] |
| *M. yunensis* (Ma, 1998) Erias et al., 2005 (syn. *Myxosoma barbodesi* Ma in Chen et Ma, 1998 | *Hypsibarbus wetmorei* (Smith, 1931) (syn. *Barbodes daruphani* Smith, 1934 ) | Gills | China | 8.0–9.6 (8.8) | 7.2 | 5.6 | 4.0–5.2 (4.7) | 2.8–3.2 (2,9) | pyrirom | — | — | 0.195—0.228 mm by 0.163—0.195 mm | [23] |

[a] Abbreviation: SL, spore length; SW, spore width; ST spore thickness; PCL, polar capsule length; PCW, polar capsule width; SF, spore form; IP, intercapsular projection; NT, number of coil turns (polar tubles). All measurements are expressed in micrometers (µm) unless otherwise stated. Ranges are presented, with the means in parentheses. For *M. flavus*, means and standard variation are shown in parentheses.
